# Antioxidant Effect of a Polyphenol-Rich Murtilla (*Ugni molinae* Turcz.) Extract and Its Effect on the Regulation of Metabolism in Refrigerated Boar Sperm

**DOI:** 10.1155/2019/2917513

**Published:** 2019-06-03

**Authors:** Ignacio Jofré, Magdalena Cuevas, Leticia Signori de Castro, João Diego de Agostini Losano, Mariana Andrade Torres, Marysol Alvear, Erick Scheuermann, André Furugen Cesar Andrade, Marcilio Nichi, Mayra Elena Ortiz Assumpção, Fernando Romero

**Affiliations:** ^1^Laboratory of Neurosciences and Biological Peptides (BIOREN-CEGIN-CEBIOR-UFRO) Medicine Faculty, Universidad de La Frontera, Francisco Salazar, 01145 Temuco, Chile; ^2^Department of Physiology, University of Concepción, PO Box 160-C, Concepción, Chile; ^3^Laboratory of Spermatozoa Biology, Department of Animal Reproduction, School of Veterinary Medicine and Animal Science, University of Sao Paulo, Avenida Prof. Dr. Orlando Marques de Paiva 87, Cidade Universitária, 05508-270 Sao Paulo, SP, Brazil; ^4^Laboratório de Andrologia, Departamento de Reprodução Animal, Faculdade de Medicina Veterinária e Zootecnia, Universidade de São Paulo, São Paulo, Brazil; ^5^Swine Research Center, School of Veterinary Medicine and Animal Science, University of São Paulo, Pirassununga, São Paulo, Brazil; ^6^Department of Chemical Sciences and Natural Resources, Faculty of Engineering and Sciences, Universidad de La Frontera, Av. Francisco Salazar, 01145 Temuco, Chile; ^7^Department of Chemical Engineering, Universidad de La Frontera, Francisco Salazar, 01145 Temuco, Chile

## Abstract

The production of reactive oxygen species (ROS) in boar spermatozoa increases in refrigeration; this can have an impact on sperm quality and fertilization capacity. We evaluated the effect of polyphenol-rich aqueous extract of murtilla (*Ugni molinae* Turcz) on boar sperm stored at 17°C in order to reduce oxidative stress and improve sperm quality in the long term. Five experiments were performed: first, characterization of the polyphenol content from five genotypes of murtilla; second, determination of the genotype with the best antioxidant effect (MT-Ex); third, the antioxidant capacity on O_2_^−^ and lipid peroxidation; fourth, the influence of MT-Ex on motility, calcium movement, cAMP, and metabolic parameters; and fifth, analysis of long-term refrigeration. The average phenolic content was 344 ppm; gallic acid, catechin, quercetin, myricetin, and kaempferol were detected. All extracts evaluated presented a concentration-dependent antioxidant effect. MT-Ex reduces intracellular O_2_^−^/peroxides but low lipid peroxidation. MT-Ex in nonstimulated ROS conditions reduces sperm motility, mitochondrial membrane potential, cAMP, and ATP, but the succinate dehydrogenase activity remained normal; also, we observed a reduction in calcium movement in *in vitro* sperm capacitation. The long-term analyses showed that MT-Ex improved sperm motility decay and reduced membrane damage and ROS at 168 h. Based on this study, we propose MT-Ex as a supplement in semen extenders.

## 1. Introduction

The world pork market grows larger every year. Pork is considered a good and cheap alternative source of animal protein and a rich source of B complex vitamins. In the swine reproduction industry, artificial insemination (AI) is the best system for increasing the number of animals and decreasing the interval between generations, a combination of factors which improves breeding programs. One of the most used systems in the swine industry is the refrigeration of semen dosage and it is recommended to maintain sperm viability during transportation/storage, and its efficiency is determined by many factors including the medium, the semen extender used, and the refrigeration time and temperature (usually 17°C) [1]. Nevertheless, this process leads to a decrease in fertility rates due to reduced sperm motility and viability [2] and increased production of reactive oxygen species (ROS) [3–5]. The purpose of an extender is to maintain the stability of the spermatozoa during the refrigeration period, reducing the number of dead spermatozoa as a result of cold stress and osmotic variations [6]. In this sense, the study of oxidative stress in boar semen allows evaluating the effects of antioxidants in the long term unlike other biological models.

Boar spermatozoa have been described as very sensitive to thermal shock because the membrane has a high content of polyunsaturated fatty acids [7]. In addition, low temperatures (9-15°C) generate a decrease in membrane phospholipid phases and damage the membrane proteins, affecting sperm viability [8] and increasing their sensitivity to lipid peroxidation. ROS have physiological functions at low concentrations, such as sperm activation [9], hyperactivation [10], and acrosome reaction [11]; at high levels, however, they may activate capacitation and reduce motility [12], generating oxidation and DNA fragmentation [13].

Antioxidants are compounds that control the effects of ROS and protect the cells against massive oxidation. Nonenzymatic antioxidants reduce ROS and indirectly protect the membrane from lipid peroxidation [14, 15]; these antioxidants, which may be found in extracellular milieux [15], can neutralize the hydroxyl radical and superoxides and prevent sperm agglutination [16]. Studies have demonstrated that supplementation of the semen extender with antioxidants (enzymatic and nonenzymatic), such as *α*-tocopherol, butylated hydroxytoluene, superoxide dismutase, catalase, cysteine, and glutathione, generates an improvement in boar sperm quality [17–21]. Molecules found in berries containing high concentrations of phenols and anthocyanin have been described as potential reducing agents against oxidative stress under *in vitro* conditions. Murtilla or murta (*Ugni molinae* Turcz.), a plant of the Myrtaceae family, is endemic to Chile and Argentina; its fruit and leaves are rich in phenolic components [22–26]. Some reports indicate that these flavonoids have a broad antioxidant activity, intervening in the signalling in cancer, cardiovascular, neuroprotection, and anti-inflammatory pathways [27]. The aims of the present work were to compare the antioxidant effect of the extracts from five different murtilla genotypes; to select the genotype with the best antioxidant capacity; to evaluate the influence of murtilla extract (MT-ex) on sperm parameters such as ATP/cAMP content, SDH activity, and ROS production; and to explore its long-term effect at 196 h, this mechanism being still unknown.

## 2. Materials and Methods

### 2.1. Bioethics Procedures and Ethics Statement

This study was carried out at the Center of Biotechnology in Reproduction, Faculty of Medicine, and the Scientific and Technological Bioresource Nucleus of La Frontera University, Chile (CEBIOR-BIOREN-CEGIN-UFRO). The study and the experimental methods were approved by the Scientific Ethics Committee (CEC) of La Frontera University and performed under the guidelines and regulations of this entity (Protocol 102/13, Acta N80/2013).

### 2.2. Reagents

Folin-Ciocalteu, Na_2_CO_3_, FeSO_4_, gallic acid, formic acid, acetonitrile, H_2_O_2_, [Fe(CN)_6_]^3−^, and NaOH were obtained from Merck. Luminol (3-aminophthalhydrazide, 5-amino-2,3-dihydro-1,4-phthalazinedione) and ATP were obtained from Sigma-Aldrich. Phosphate-buffered saline (PBS), Hank's buffered saline solution, and all fluorescent dyes were obtained from Thermo Fisher.

### 2.3. Plant Material and Extraction Procedure

Fresh fruits of five genotypes of murtilla (*Ugni molinae* Turcz.) (G14-4, G19-1, G22-1, G23-1, and G27-1) were obtained from the Agricultural Research Institute, Vilcún, Chile (INIA Carillanca). The fruits were harvested in April 2012 at the INIA Carillanca experimental station located close to Puerto Saavedra, Araucanía Region, Chile (38°45′S, 73°21′W). Six grams of fruit of each genotype was ground in a mortar and transferred to a bottle containing 20 mL^−1^ of prewarmed (30°C) distilled water. The mixture was shaken in an incubator (GFL 3032, Germany) at 170 rpm, 30°C, for 20 min and vacuum-filtered (Whatman N° 1 filter paper). The aqueous extract was stored under refrigeration, protected from light and oxygen, until each analysis.

### 2.4. Experiment 1: Chemical Characterization

#### 2.4.1. Determination of Total Polyphenol Content

The total polyphenol content was determined using the Folin-Ciocalteu method as described by Alfaro et al. [28]. Aqueous murtilla fruit extract (40 *μ*L) was mixed with distilled water (3.16 mL) in a test tube, and then 200 *μ*L of Folin-Ciocalteu reagent was added. After 5 min at 20°C, 600 *μ*L of 20% Na_2_CO_3_ was added to the reaction mixture, which was maintained at 20°C for 120 min in the dark. The absorbance was measured at 765 nm using a spectrophotometer (Spectronic Genesys 5, Sweden), and the results were expressed as *μ*g of gallic acid equivalent per mL (*μ*gGAE mL^−1^) of aqueous extract.

#### 2.4.2. Phenolic Compound Concentration by HPLC Analysis

Phenolic compounds were identified from the five murtilla genotypes using the Merck Hitachi High-Performance Liquid Chromatography system (LaChrom, Tokyo, Japan) coupled to an L-7100 pump and L-4250 UV-VIS detector. A 5 *μ*m C18 RP Inertsil ODS-3 column (GL Sciences Inc., Tokyo, Japan) was used with a 250 mm × 4.60 mm i.d., maintained at 25°C. The original extract was concentrated to dryness using a rotary evaporator (Büchi R-210, Germany) at 140 rpm, 30°C, and then resuspended in 5 mL^−1^ of methanol : formic acid (99:1, *v*/*v*). The sample extract was filtered through a 0.45 *μ*m filter, and 20 *μ*L was injected for polyphenol analysis. The identification of compounds was confirmed both by comparison of their retention time with pure standards and by coinjection. A linear gradient solvent system consisting of 1% formic acid (A) and acetonitrile (B) was used at a flow rate of 1 mL^−1^ min as follows: 0-2 min, 100% A; 2-15 min, 80% A/20% B; 15-20 min, 70% A/30% B; 20-30 min, 40% A/60% B; and 30-35 min, 100% A. Phenolic compounds were detected at 280 nm [28].

### 2.5. Experiment 2: Antioxidant Capacity of the Extracts

The second experiment was carried out in two assays: (1) antioxidant screening to determine which of the 5 murtilla genotype extracts presented the best antioxidant capacity in refrigerated sperm samples stored in the MR-A semen extender and (2) determining the influence of time and concentration of antioxidant response under refrigeration (17°C), to reveal the maximum and minimum time limits to induce the antioxidant effect using a mathematical model to explain the antioxidant behaviour of the murtilla genotype extract selected in the first assay (MT-Ex).

#### 2.5.1. Semen Processing

Sperm samples from ten boars (5 collections each) aged 15 to 28 months from Sociedad Agrícola y Ganadera Pehuén Ltda. (Victoria, Chile) were used in experiments 2, 3, and 4. The sperm samples were collected weekly from each boar using the gloved-hand technique. Semen samples were purified, and the sperm-rich fraction was placed in an MR-A (Minitube®) semen extender prewarmed to 37°C; the concentration was adjusted to 25 × 10^6^ cells/mL and refrigerated at 17°C. In attempt to normalize the possible individual variations, we performed seminal analysis of fresh ejaculate from the 10 boars, which included total motility (>70%), concentration, and morphology (<20% abnormal spermatozoa), and according to the parameters established by the Brazilian College of Animal Reproduction, all of them were approved by these criteria.

#### 2.5.2. Oxidative Stimulation in Boar Sperm

The ideal oxidative stimulation condition was performed with H_2_O_2_ as a prooxidant. The stimulation was performed with 122.7 *μ*M, during 6 h of incubation according to a standard curve of stimulation (data not shown). All stimulated samples were compared with a control without stress stimulation (without H_2_O_2_).

#### 2.5.3. Antioxidant Screening

For antioxidant screening, we used the luminol assay [29] modified which evaluates oxidation in the whole sample, where in the presence of luminol and iron the sample generates a luminescence directly proportional to the oxidation status. Samples with 25 × 10^6^ cells (1 mL of final volume) were incubated with 122.7 *μ*M of H_2_O_2_ for 6 h to stimulate the oxidant condition; then, the samples were washed by centrifugation (300g, 10 minutes) to remove the H_2_O_2_ in excess and diluted in 1 mL of MR-A. Concentrations of the five different murtilla genotypes were then added (0.0001 to 100 *μ*gGAE mL^−1^), and the samples were incubated for 6 h at 17°C. At the end of the incubation period, each reaction (150 *μ*L) was centrifuged at 700g for 10 min, the supernatant was discarded, and the pellet was suspended in 300 *μ*L of Luminol (600 *μ*M in PBS 1x) and incubated for 15 min at 37°C in a Luminometer microplate reader (Luminoskan, Thermo Scientific). Then, 25 *μ*L of the developed solution was added using an automated dispenser (0.1 M of [Fe(CN)_6_]^3−^ in 0.1 M of NaOH, pH 8) and immediately measured with an integration of 1000 milliseconds (ms). The data were compared with a control treated with H_2_O_2_ and without antioxidant treatment. Transformation of the *X*-axis to log_10_ was performed followed by a nonlinear regression (dose response) to determine the EC50 (effective concentration to reduce stress by 50%) of the antioxidant effect of each extract evaluated. The extract which presented the lowest dispersion of data was selected as the best antioxidant; subsequent experiments were executed with the murtilla extract selected, G14-4, hereafter “MT-Ex”.

#### 2.5.4. Time and Concentration Effect of MT-Ex

The method was performed using 25 × 10^6^ cells in MR-A semen extender. Each sample was incubated with 122.7 *μ*M of H_2_O_2_ for 6 h at 17°C to stimulate ROS production, then centrifuged at 700g for 10 min. The resulting pellets were resuspended in the MR-A semen extender supplemented with 0.0001–100 *μ*gGAE mL^−1^ of MT-Ex and incubated for 30 min to 6 h at 17°C. The antioxidant effect was evaluated following the Luminol protocol described above.

### 2.6. Experiment 3: Effect of MT-ex on Oxidative Stress and Sperm Motility

To detect the superoxide anion and lipid peroxidation, we used 25 × 10^6^ cells in the MR-A semen extender, divided into 4 groups: “control”—sperm samples incubated in the MR-A semen extender, “oxidant”—sperm samples treated with an oxidant agent, “MT-Ex”—sperm samples treated with 0.0315 *μ*gGAE mL^−1^ as antioxidant treatment (concentration obtained in experiment 2), and “MT-Ex + oxidant”—sperm samples coincubated with oxidant agent and 0.315 *μ*gGAE mL^−1^ as antioxidant treatment. The oxidant agent used was 122.7 *μ*M of H_2_O_2_ to stimulate superoxide anion/hydrogen peroxide production and 100 *μ*M of FeSO_4_ to stimulate lipid peroxidation. All groups were incubated for 30 minutes at 17°C.

#### 2.6.1. Intracellular Superoxide-Anion Reduction

The capacity of the selected extract to reduce superoxide anion and hydrogen peroxide was measured using dihydroethidium (DHE) as a fluorescent indicator of ROS in combination with SYTOX Green as a membrane damage indicator [30]. At the end of incubation, all groups were washed using MR-A with prewarming and centrifuged for 10 minutes at 900g. Staining was performed with 2.2 *μ*M of DHE and 0.04 *μ*M of SYTOX Green for 20 minutes at 37°C in the MR-A semen extender. At the end of incubation, the samples were suspended in 300 *μ*L of prewarmed PBS (1x) and immediately analysed in a flow cytometer (FACSCanto II, BD) using the spectra 488/583 (DHE) and 488/523 (SYTOX Green) analysing 10,000 events per sample. The data was analysed in Flowing Software 2.0, gating the SYTOX Green negative events (viable cells); the population was then assessed with high DHE fluorescence (viable cells with high ROS production).

#### 2.6.2. Lipid Peroxidation

Membrane lipid peroxidation was determined using the BODIPY C-11 fluorescent sensor, which exhibits a red basal fluorescence in the nonoxidized state, while oxidation shows a green fluorescence. The method was carried out according to Aitken et al. with some modifications [31]. Thirty minutes before oxidant and antioxidant treatment, the sperm samples were incubated with 100 nmol of BODIPY C-11 in MR-A for 30 minutes at 37°C. The samples were washed twice with prewarmed MR-A and centrifuged at 700g for 10 minutes to remove unbound BODIPY C-11 residues. The same samples were divided into the 4 treatment groups described above. At the end of treatment incubation, each sample was washed with PBS 1x and immediately analysed in a microplate reader (Synergy HT, BioTek) to detect the mean fluorescence using the wavelength 488/590 for the nonoxidized fraction and 488/530 for the oxidized fraction according to Drummen et al. [32].

### 2.7. Experiment 4: Effect of Murtilla Extract on Sperm Metabolism

The same groups described in experiment 3 were used in this experiment. We characterised sperm motility, succinate dehydrogenase activity, mitochondrial membrane potential, and ATP content.

#### 2.7.1. Sperm Motility

Sperm motility evaluation was performed in two steps: first, an assay to determine the concentration effect of MT-Ex on sperm motility. The MR-A semen extender was supplemented with 0 to 10 *μ*gGAE mL^−1^, then 25 × 10^6^ cells/ml were added to the solution and incubated for 30 minutes at 17°C. The second step was carried out with the same groups described in experiment 3. In both cases, the temperature was increased to 37°C five minutes before the end of incubation to recover sperm motility. Two microliters of sample was loaded in a slide chamber (Leja), and the motile, static, and progressive populations were analysed (%) in Automatic Sperm Analyzer System according to the following parameters: 45 frames at a frame rate of 60 frames/s; minimum contrast = 46; minimum cell size = 7 pixels; motility > 45 *μ*m/s; progressive motility > 45 *μ*m/s; and straightness > 45%.

#### 2.7.2. Mitochondrial Membrane Potential (MMP)

The mitochondrial membrane potential was evaluated by a JC-1 probe [33] determining the sperm population with basal mitochondrial membrane potential (MMP) in oxidant and antioxidant conditions. At the end of the treatment, 2 × 10^5^ cells were incubated with 76.6 *μ*g/mL of JC-1 and 0.5 *μ*M propidium iodide for 5 minutes at 37°C. At the end of incubation, the cells were suspended in 300 *μ*L of prewarmed PBS (1x) and immediately analysed in a flow cytometer (FACSCanto II BD) analysing 10,000 events per sample.

#### 2.7.3. Succinate Dehydrogenase Activity and MTT Reduction Assay

The succinate dehydrogenase activity assay was based on reduction of tetrazolium salts to insoluble formazan in the presence of succinate by the mitochondrial enzyme succinate dehydrogenase, which allowed the metabolic rate related to aerobic respiration to be determined [34]. Each sample of intact cells was incubated with MTT (3.5 *μ*g/mL) for 1 hour at 37°C; at the end of incubation, 100 *μ*L of DMSO was added to lysate cells, the formazan crystals were solubilized, and the reaction was stopped. The colorimetric reaction was measured in a microplate reader using an absorbance of 514 nm (Synergy HT, BioTek). The data was compared with the nontreated group (100% normal enzymatic activity).

#### 2.7.4. ATP Content

The influence of MT-Ex on ATP content was measured using a luminescence-dependent indicator (CellTiter, Promega) [35]. At the end of incubation, 100 *μ*L of each sperm sample (5 × 10^5^ cells) was incubated with 100 *μ*L of the ATP probe mixture following the manufacturer's recommendations. The solution was incubated for 10 minutes in a shaker (180 rpm), and the luminescence was immediately measured in a luminometer (Luminoskan, Thermo Scientific) with integration of 1000 ms. The luminescence measured was compared with the standard curve for ATP (0.0001 to 1000 nmol), based on a linear regression, and the data were interpreted as nmol of ATP in 1 × 10^6^ cells.

#### 2.7.5. cAMP and Calcium Movement

cAMP was determined using the commercial kit cAMP-Screen Direct (Invitrogen) [36]. At the end of incubation, the sperm samples were centrifuged at 700g for 10 minutes and washed with HBSS 1x (Ca^2+^- and Mg^2+^-free). cAMP was measured following the manufacturer's instruction for suspended cells. The data were compared with a standard curve for cAMP (0.0006 to 6000 pmol), analysed by sigmoidal equation, and interpreted as pmol of cAMP in 1 × 10^6^ cells.

The transient calcium was measured to determine the effect of MT-Ex on the capacitation state of sperm samples. The measurement method used was the green fluorescent probe FLUO-4 AM (Invitrogen) to detect the calcium signal [37]. First, buffers were prepared to stimulate sperm capacitation following Supplementary Table S1. Before stimulation, the sperm samples (25 × 10^6^ cells) were incubated with 1 *μ*M of FLUO-4 AM (without pluronic acid F-127) for 45 minutes at 37°C in the MR-A semen extender. The samples were divided into 4 groups and washed twice (700g, 10 minutes) to remove the unbound stain. Each group was diluted in each prepared buffer and maintained at 17°C until analysis. Then 15 × 10^6^ cells were placed in a microplate (with optical glass bottom), and the fluorescence was recorded for 50 min at 60-second intervals using a microplate reader (BioTek Synergy HT) in the 488/523 spectrum.

#### 2.7.6. TUNEL Assay

The In Situ Cell Death Detection kit (Roche, Mannheim, Germany), which is based on the terminal deoxynucleotidyl transferase- (TdT-) mediated dUTP nick-end labeling (TUNEL) technique, was used to analyse the sperm DNA fragmentation. At the end of the incubation, 5 × 10^6^ cells were recuperated of each treatment and incubated with 2 mmol^−1^ of dithiothreitol (1 mL final volume) for 45 min at room temperature in order to relax the chromatin and allow the enzyme better access to the DNA [38]. Subsequently, the cells were washed twice with PBS, fixed in 2% paraformaldehyde for 15 min at 4°C, and permeabilized with 0.1% Triton X-100 for 10 min at room temperature. Then, the sperm were incubated with the TUNEL reaction solution and incubated for 1 h at 37°C. Finally, the cells were washed twice, resuspended in DPBS, and analyzed by flow cytometry. The results were expressed as the percentage of FITC-positive cells.

### 2.8. Experiment 5: Long-Term Refrigeration

In this experiment, long-term analyses were performed to evaluate sperm motility, viability, mitochondrial membrane potential, and ROS and O_2_^−^ production. The samples were divided into two groups: control—semen diluted in the MR-A semen extender; MT-Ex—semen diluted in the MR-A semen extender and supplemented with 0.315 *μ*gGAE mL^−1^ of MT-Ex. Both groups were refrigerated at 17°C for 168 h and evaluated at 24 h intervals.

#### 2.8.1. Semen Processing

Five mature boars (*n* = 15; 5 animals × 3 collections), aged 15 to 28 months, were used in this study; they were obtained from the Swine Research Center, School of Veterinary Medicine and Animal Science, University of São Paulo, Pirassununga, São Paulo, Brazil. The sperm samples were collected weekly from each boar using the gloved-hand technique. Semen samples were purified, and the sperm-rich fraction was placed in the MR-A extender supplemented with 0.315 *μ*gGAE mL^−1^ prewarmed to 37°C and then refrigerated at 17°C (25 × 10^6^ cells/ml). In attempt to normalize the possible individual variations, we performed seminal analysis of fresh ejaculate from the 10 boars, which included total motility (>70%), concentration, and morphology (<20% abnormal spermatozoa), and according to the parameters established by the Brazilian College of Animal Reproduction, all of them were approved by these criteria.

#### 2.8.2. Sperm Motility

Motility parameters were evaluated using the Computer Assisted Sperm Analysis (CASA) system (IVOS, v. 12.2, Hamilton Thorne Research, Beverly, MA). The parameters used were 45 frames at a frame rate of 60 frames/s; minimum contrast = 46; minimum cell size = 7 pixels; motility > 45 *μ*m/s; progressive motility > 45 *μ*m/s; and straightness > 45%. In brief, each slide was heated to 37°C; 7 *μ*L of the sample was placed in the slide and covered with a coverslip. A minimum of six fields were selected for analysis. The data obtained to observe differences between control and MT-Ex were the total motile population.

#### 2.8.3. Sperm Characteristics

Aliquots of 2 × 10^5^ cells were obtained every 24 h to evaluate mitochondrial membrane potential (JC-1), and production of superoxide anion/hydrogen peroxides (DHE) following the procedures described above. One extra protocol was performed in this experiment, to determine ROS production: determination of total ROS by CellROX Green/PI [39]. Aliquots of 2 × 10^5^ cells were stained with 5 *μ*M of CellROX Green for 30 minutes at 37°C; in the last 10 minutes, PI was added to a final concentration of 6 *μ*M. All fluorescent reactions were performed in a Guava easyCyte flow cytometer (Merck Millipore) analysing 10,000 events per sample using a 488 laser and fluorescence channels according to the manufacturer's recommendations.

### 2.9. Statistical Analysis

For experiment 1, the data were analysed using one-way analysis of variance (ANOVA). The values obtained at *P* < 0.05 were considered significant. The differences between means were determined using Tukey's multiple comparison tests. The results were expressed as the means of the measurements and their corresponding standard deviations. For experiment 2, the concentration-response analysis was performed by polynomial regression to determine EC50. The EC50 obtained for each genotype was subjected to analysis of variance (ANOVA), and the significance of the difference between means was determined by Tukey's test (*P* < 0.05). The Time and Concentration analysis was evaluated by ANOVA to determine the interaction between variables. Once the absence of the interaction between variables was verified, a prediction analysis of the surface plot and equation that describes the mathematical model was carried out using the software Curve Expert (Microsoft 2.2). The equation was solved by log transformation of the concentration parameter and full cubic polynomial regression. The EC50 analysis for each time was performed by polynomial regression using GraphPad Prism. For experiment 3, evaluation of O_2_^−^/peroxide production and lipid peroxidation was subjected to analysis of variance (ANOVA) and the significance of the difference between means was determined by Tukey's test (*P* < 0.05). For experiment 4, sperm motility data were analysed by log transformation of the concentration variable and sigmoidal nonlinear regression. The mitochondrial membrane potential, SDH activity, ATP, and cAMP content were subjected to analysis of variance (ANOVA), and the significance of the difference between means was determined by Tukey's test (*P* < 0.05). Calcium analysis was performed by linear regression comparing the linear trends. For experiment 5, long-term analysis was performed by calculation of means and S.D., and statistical analysis of the results was performed using GraphPad Prism 5.0. Analysis of repeated measures of variance was done to compare semen motility, mitochondrial membrane potential, viability, ROS production, and mitochondrial O_2_^−^ between the control and MT-Ex groups and over time, followed by a Bonferroni significant difference test (to locate differences). Values were considered significant when *P* < 0.05.

## 3. Results

### 3.1. Experiment 1: Chemical Characterization

The total polyphenol content in MT-Ex ranged from 85.5 to 406.5 *μ*gGAE mL^−1^; the highest value was obtained from genotype 19-1 (Table 1). Phenolic compounds identified by HPLC analysis were gallic acid, catechin, quercetin-3-*β*-D-glucoside, myricetin, quercetin, and kaempferol; their concentrations for the five genotypes are shown in Table 1. The flavonol catechin was the most abundant constituent in our samples with 2.69, 0.521, 10.01, 2.28, and 1.81 *μ*g/mL in the G14-4, G19-1, G22-1, G23-1, and G27-1 genotypes, respectively. The concentrations of gallic acid, quercetin-3-*β*-D-glucoside, myricetin, quercetin, and kaempferol in MT-Ex ranged from 0.009 to 3.21 *μ*g/mL.

### 3.2. Experiment 2: Extract Antioxidant Capacity

#### 3.2.1. Antioxidant Screening

**Figure 1:**
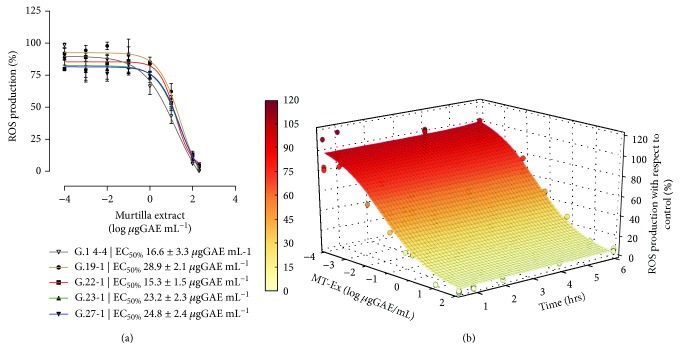
Antioxidant activity of MT-Ex. (a) Extracellular antioxidant effect of five genotypes of MT-Ex (log of 0.0001 to 100 *μ*gGAE mL^−1^) in sperm cells pretreated with 122.7 *μ*M of H_2_O_2_ as oxidant agent. The EC50 for each genotype indicates the effective concentration to reduce oxidative stress by 50%. (b) Time and concentration evaluation of antioxidant activity of murtilla genotype 14-4 8 (MT-Ex) in sperm samples pretreated with H_2_O_2_ as oxidant treatment.

We evaluated the antioxidant potential of fruit extract from five *Ugni molinae* genotypes, using H_2_O_2_ as prooxidant agent to reveal the minimum and maximum concentrations of antioxidant effect. In all genotypes evaluated, the results showed a concentration-dependent effect when the antioxidant concentration increased (Figure 1(a)). We observed similar tendencies in the sigmoidal curves and EC50 calculated for each genotype: 16.6 *μ*gGAE mL^−1^ (G14-4), 28.9 *μ*gGAE mL^−1^ (G19-1), 15.3 *μ*gGAE mL^−1^ (G22-1), 23.2 *μ*gGAE mL^−1^ (G23-1), and 24.8 *μ*gGAE mL^−1^ (G27-1). The average of the five genotypes was 21.76 *μ*gGAE/mL^−1^. Genotype G14-4 was selected as the ideal extract in the characterization and antioxidant analysis because of the low dispersion of the data in the curve (*R* ^2^: 0.995). The results below were performed using only G14-4, hereafter “MT-Ex”.

#### 3.2.2. Time and Concentration Effects of MT-Ex

Evaluation of the surface plot revealed that the effect was concentration-dependent (Figure 1(b)) and partially time-dependent. This profile is explained in a mathematical model according to the calculated equation (1) where *x*1 corresponds to time (in hours) and *x*2 to the log of the concentration in *μ*gGAE mL^−1^.

(1) $$\begin{array}{c} y=22.09-16.79x1+3.91x2+3.43x1^{2}+0.2x2^{2}+0.72x1^{3}+0.05x2^{3}. \end{array}$$

The EC50 calculated for each hour showed an effect indirectly proportional to the time of antioxidant treatment: the EC50 values at 30 minutes and 1, 2, 4, and 6 h were 0.315, 0.055, 0.030, 0.003, and 0.006 *μ*gGAE mL^−1^, respectively (Table 2). We determined that the optimum concentration and time, with low variation of effective concentration for the increase in exposure time, was 0.315 *μ*gGAE mL^−1^ for 30 minutes. This combination was used in subsequent analyses.

### 3.3. Experiment 3: Effect of Murtilla Extract on Sperm Oxidative Stress Parameters

#### 3.3.1. Intracellular Superoxide-Anion Reduction

**Figure 2:**
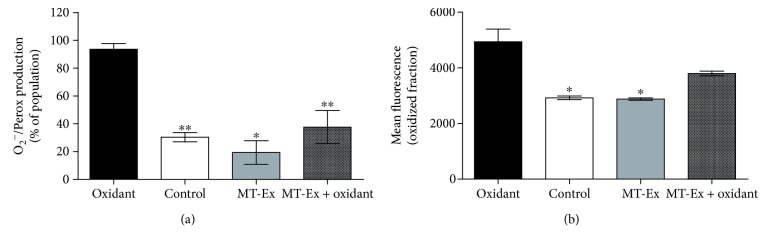
Antioxidant effect of MT-Ex. (a) Antioxidant capacity of MT-Ex on peroxides and superoxide anion production. (b) Effect of MT-Ex on lipid peroxidation. In both analyses: “control”—sperm samples incubated in MR-A semen extender; “oxidant”—sperm samples treated with oxidant agent; “MT-Ex”—sperm samples treated with 0.0315 *μ*gGAE mL^−1^ as antioxidant treatment; and “MT-Ex + oxidant”—sperm samples coincubated with oxidant agent and 0.315 *μ*gGAE mL^−1^ as antioxidant treatment. The oxidant agents were H_2_O_2_ and FeSO_4_ to produce O_2_^−^/perox and lipid peroxidation, respectively. Differences observed were ^∗^ *P* < 0.05 and ^∗∗^ *P* < 0.01 with respect to the “oxidant” group.

The EC50 which produced an antioxidant effect (0.315 *μ*gGAE mL^−1^ for 30 min) on O_2_^−^ production was evaluated by the DHE probe. As expected, the oxidant group presented the highest percentage of population with high O_2_^−^ production (93.6% ± 4.1). This was followed by the MT-Ex + oxidant group (37.67% ± 5.6) and the control (30.3% ± 3.2) with no statistical difference between them. The lowest percentage (statistically significant difference) of viable cells with high superoxide production (19.3% ± 4.9) was found in the antioxidant group (MT-Ex) (Figure 2(a)).

#### 3.3.2. Lipid Peroxidation

Lipid peroxidation was evaluated by the BODIPY C-11 probe; we observed a different response profile between treatment groups when compared to superoxide-anion reduction. In this case, the oxidant and MT-Ex + oxidant groups presented the highest fluorescence intensity (4938 ± 455.2 and 3796 ± 147.2), while the control and MT-Ex groups presented lower fluorescence intensities (2921 ± 64.6 and 2879 ± 44.4, respectively) (Figure 2(b)).

### 3.4. Experiment 4: Effect of MT-Ex on Sperm Metabolism

#### 3.4.1. Sperm Motility

**Figure 3:**
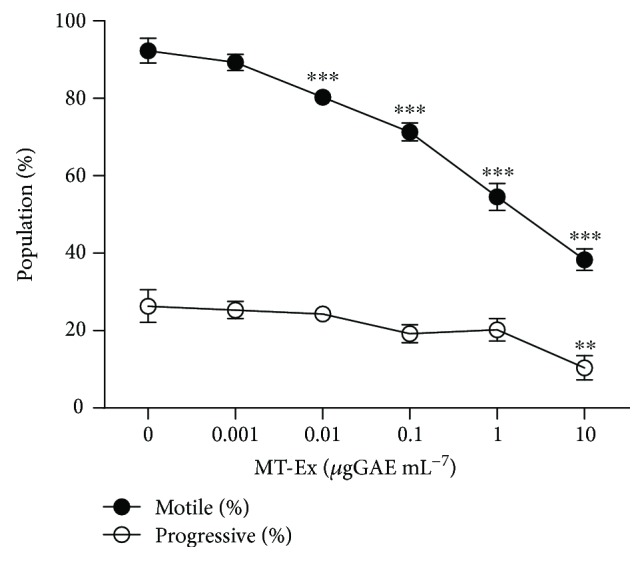
Effect of MT-Ex on sperm motility. Analysis of motile, progressive, and static population treated with MT-Ex for 30 min. A sigmoidal representation of the effect of different concentrations of MT-Ex on sperm motility. Samples treated with MT-Ex were stored at 17°C with MT-Ex and sperm motility was triggered by increasing the temperature to 37°C. Differences observed were ^∗^ *P* < 0.05 and ^∗∗^ *P* < 0.01 with respect to the “0” group.

The effect of MT-Ex on sperm motility was evaluated in two steps. First, we examined the effect of increasing concentrations of MT-Ex on sperm motility during refrigerated incubation: we observed a concentration-dependent decrease in total motility and a consequent increase in static cells, without any significant change in the progressive population (Figure 3).

In the second trial, we determined the influence of MT-ex on sperm motility in oxidant and antioxidant conditions. A reduction in sperm motility was observed in all groups treated. As expected, the oxidant group resulted in a reduction in the motile sperm population from 89.6% ± 3.1(control) to 56.3% ± 2.6 (oxidant); however, treatments with MT-Ex on cells without oxidative stimulation resulted in a reduction in sperm motility (65.3% ± 1.5), while the addition of MT-Ex to oxidized sperm did not improve the motility parameter; on the contrary, a substantial reduction was observed with respect to the oxidant group (46.3% ± 1.9) (Table 3).

### 3.5. Mitochondrial Membrane Potential (MMP)

**Figure 4:**
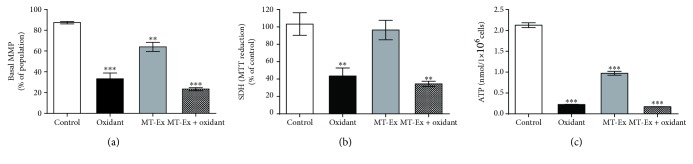
Analysis of metabolic parameters in sperm samples treated with MT-Ex. (a) Effect of MT-Ex on mitochondrial membrane potential. The data shown represent the cell count with normal potential measured by the JC-1 probe. (b) Succinate dehydrogenase activity evaluated by MTT assay. (c) Determination of ATP content. The groups were “control”—sperm samples incubated in the MR-A semen extender, “oxidant”—sperm samples treated with oxidant agent, “MT-Ex”—sperm samples treated with 0.0315 *μ*gGAE mL^−1^ as antioxidant treatment, and “MT-Ex + oxidant”—sperm samples coincubated with oxidant agent and 0.315 *μ*gGAE mL^−1^ as antioxidant treatment. Significant differences are represented between control and groups. ^∗^ *P* < 0.05, ^∗∗^ *P* < 0.01, and ^∗∗∗^ *P* < 0.001.

The mitochondrial membrane potential was evaluated with the JC-1 probe as a measure of the influence of MT-Ex on spermatozoa in oxidant and antioxidant conditions. We observed that the cell count with basal MMP in the control group was the highest with 88.6%. The addition of H_2_O_2_ as an oxidative stimulator reduced this parameter, independently of the presence or absence of MT-Ex, with similar results between groups (34.1% and 32.7%, respectively). However, the addition of MT-Ex in nonoxidized sperm presented a higher percentage of basal MMP (65.7%) when compared to groups with H_2_O_2_, but still lower than in the control group (Figure 4(a)).

### 3.6. Succinate Dehydrogenase and MTT Reduction Assay

The evaluation of succinate dehydrogenase activity showed that the presence of MT-Ex resulted in no significant differences compared to control (96.3%). However, for the oxidant group, the percentage of cells was reduced to 41.5%, while in the MT-Ex + oxidant group, the MMP did not present substantial improvements (Figure 4(b)).

### 3.7. ATP Content

The effect of MT-Ex on ATP content was evaluated under different incubation conditions. We observed a reduction in ATP content in all treatments under oxidant and antioxidant conditions. The ATP content in the group treated with MT-Ex without oxidant stimulation was reduced from 2.29 ± 0.61 nmol (control group) to 1.06 ± 0.23 nmol (MT-Ex group). On the other hand, the ATP contents in the groups treated with H_2_O_2_ (oxidant) and with MT-Ex and H_2_O_2_ (MT-Ex + oxidant) were reduced by 0.20 ± 0.03 nmol and 0.23 ± 0.04 nmol, respectively (Figure 4(c)).

## 4. cAMP and Calcium Movement

**Figure 5:**
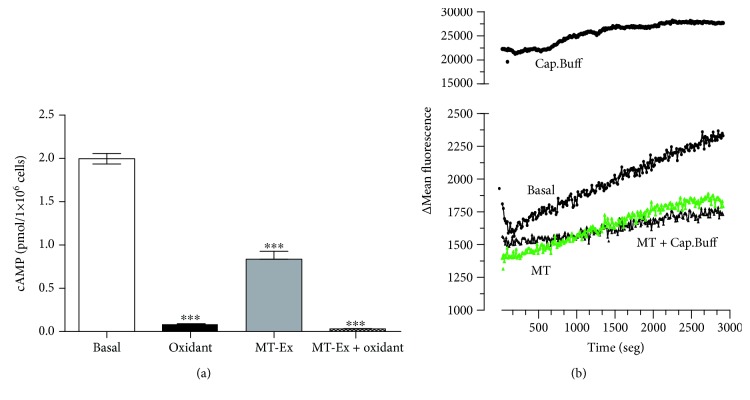
Analysis of cAMP and calcium movement of sperm samples treated with MT-Ex. (a) cAMP content of sperm samples in oxidant and antioxidant conditions. (b) Evaluation of calcium release of sperm samples in different incubation conditions. Basal: TALP-HEPES; Cap.Buffer: capacitating buffer (CaCl_2_+HCO_3_); MT: TALP-HEPES + 10 *μ*gGAE mL^−1^ of MT-Ex; MT+Cap.Buffer: capacitating buffer + 10 *μ*gGAE mL^−1^ of murtilla. Significant differences are represented between basal and groups. ^∗^ *P* < 0.05, ^∗∗^ *P* < 0.01, and ^∗∗∗^ *P* < 0.001. *N* = 5.

We evaluated the effect of MT-Ex and H_2_O_2_ on the cAMP content of sperm samples under refrigeration and subsequently the influence of MT-Ex on calcium movement. The results suggest a reduction in the cAMP content in all the treated groups: in oxidant conditions, the cAMP concentration was reduced, reaching 0.19 pmol ± 0.03, while in the MT-Ex group the concentration was 0.95 pmol ± 0.21. However, treatment with MT-Ex did not improve the loss of cAMP (Figure 5(a)) in oxidized cells.

In order to corroborate the effect of cAMP as a physiological indicator of important processes in capacitation and acrosomal reaction, we evaluated the intracellular calcium movement of sperm samples maintained in different buffers. The results suggest that the fluorescent signal of FLUO-4 AM increased in sperm samples in the capacitation buffer, indicating an increase in intracellular calcium, whereas in the basal condition without calcium stimulation, a small increase in fluorescence was observed as expected. Surprisingly, the addition of 10 *μ*gGAE mL^−1^ of MT-Ex to the capacitation buffer reduced the fluorescence of the calcium indicator significantly as compared to the sperm samples without addition of MT-Ex. The sperm samples maintained in TALP-HEPES and supplemented with MT-Ex presented a similar behaviour. For all cases, it was observed that as time increased, there was an increase in the fluorescent signal; however, the greatest change in fluorescence over time was observed in the basal condition (Figure 5(b)).

### 4.1. DNA Fragmentation

**Figure 6:**
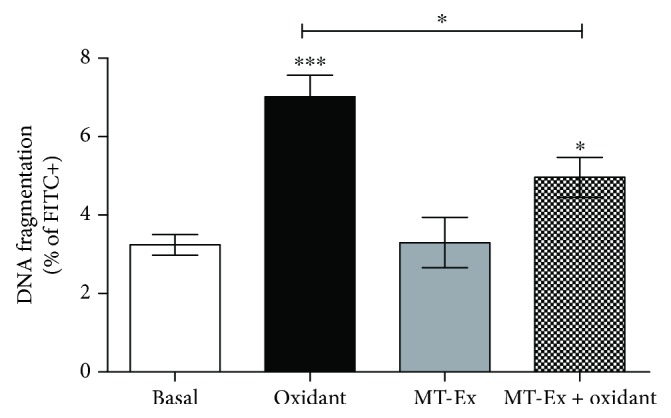
Analysis of DNA fragmentation in sperm samples treated with MT-Ex. The data shown represent the cell count with FITC + fluorescence. The groups were “control”—sperm samples incubated in the MR-A semen extender, “oxidant”—sperm samples treated with oxidant agent, “MT-Ex”—sperm samples treated with 0.0315 *μ*gGAE mL^−1^ as antioxidant treatment, and “MT-Ex + oxidant”—sperm samples coincubated with oxidant agent and 0.315 *μ*gGAE mL^−1^ as antioxidant treatment. Significant differences are represented between control and groups. ^∗^ *P* < 0.005, ^∗∗^ *P* < 0.01, and ^∗∗∗^ *P* < 0.001.

We evaluated the influence of MT-Ex on DNA fragmentation under basal and oxidant conditions. Only 3.24 ± 0.58% of the population showed DNA fragmentation in the basal group, while in oxidant conditions this parameter increased at 7.02 ± 1.21% (*P* < 0.001). The MT-Ex supplementation in nonoxidizing conditions did not show significant differences with respect to basal conditions, while the antioxidant treatment decreased the count of sperm cells with fragmented DNA with respect to the oxidant group (*P* < 0.005) (Figure 6).

### 4.2. Experiment 5: Long-Term Effect

**Figure 7:**
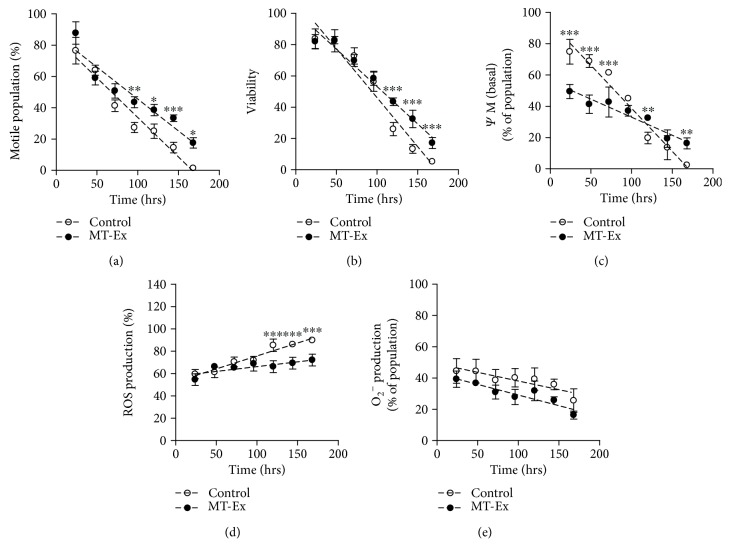
Long-term analysis. Effect of MT-Ex on sperm parameters, from 24 to 168 h of incubation at 17°C. In all analyses, white circles represent the control group—sperm samples stored in the MR-A semen extender, and black circles represent the MT-Ex group—sperm samples stored in the MR-A semen extender supplemented with MT-Ex. (a) Motile sperm population, (b) viable sperm count, (c) sperm population with basal mitochondrial membrane potential (MMP), (d) viable sperm cells with high ROS production, and (e) viable cells with high O_2_^−^/peroxide production. Significant differences are represented between control and MT-Ex. ^∗^ *P* < 0.05, ^∗∗^ *P* < 0.01, and ^∗∗∗^ *P* < 0.001.

Long-term analysis was carried out to determine the antioxidant capacity and verify the stabilizing properties of MT-Ex over time. In the study, it was observed that the addition of MT-Ex to the MR-A semen extender increased the number of motile cells at 168 h (17.5 ± 8.3%) as compared to the control (1.3 ± 1.9%). The reduction rates of sperm motility in the two groups were control 12.17% per day and MT-Ex 9.08% per day (Figure 7(a)).

The analysis of sperm viability was performed by a propidium iodide probe, which depends on membrane stability. We observed an increase in cell survival in samples with long exposure times, with significant differences observed between groups at 144 to 168 h. At the last time evaluated, the MT-Ex group presented an undamaged cell count of 17.1% ± 3.6, versus 5.2% ± 1.2 of cells with intact membrane in the control group. The reduction in viability over time was control -15.05% per day and MT-Ex -11.45% per day (Figure 7(b)).

The MMP analysis showed a higher angular coefficient of fall in the control group than in the MT-Ex group (-13.2% and -3.22% per day, respectively), indicating that populations with basal MMP are rapidly reduced over time in the absence of MT-Ex. Thus, the addition of MT-Ex to the MR-A semen extender delayed the loss of membrane potential in the last hours of the analysis (16.3% ± 3.5), in contrast to the control which presented a substantial decrease in populations with basal potential (2.4% ± 1.4) (Figure 7(c)).

Oxidative stress production was lower in the samples treated with MT-Ex than in the control. It was observed that the endogenous production of ROS in viable cells after 168 h reached 79.1% ± 5.3 in the presence of MT-Ex while the value in the control was 89.8% ± 2.6 (Figure 7(d)). ROS production increased over time in both groups, in the control group by 5.56% per day and in the MT-Ex group by 2.1% per day. Antioxidant treatment with MT-Ex reduced the normal increase in ROS production in the longest refrigeration time (168 h). On the other hand, the evaluation of mitochondrial O_2_^−^ production showed a reduction in this parameter in both groups, with no significant difference when the storage time was increased (Figure 7(e)).

## 5. Discussion

The ability of antioxidant agents to control the harmful effects of reactive oxygen species on spermatozoa has been extensively investigated, to further their use as the main regulators of these oxidant molecules. However, the main focus of the present study was on sperm quality rather than the influence of antioxidant agents on metabolic functionality and sperm physiology [40–43]. Here, for the first time we evaluated the antioxidant and metabolic effects of murtilla as an antioxidant supplement in boar MR-A semen extender in an attempt to improve sperm refrigeration based on the behaviour of sperm metabolism.

In previous studies, various genotypes evaluated presented differences in antioxidant activity measured by the DPPH test [22, 23]. These variations in the composition and concentration of phenolic compounds normally involve distinct biological effects due to synergic actions of certain compounds. The total polyphenol contents determined for murtilla fruit aqueous extract from five genotypes (G14-4, G19-1, G22-1, G23-1, and G27-1) in the present study were lower than in the murtilla fruit aqueous extract from three other locations in Chile with different climatic conditions [44]. The phenolic compounds identified (Table 2) have commonly been found in murtilla fruit and leaves [22–26, 45]. The flavonol catechin, which is the most abundant constituent, has been considered a valuable chemical marker for antioxidant activity in naturally occurring agents [44].

Boonsorn et al. reported that the addition of catechin in an extender for boar spermatozoa could improve sperm motility, viability, and acrosome integrity and reduce malondialdehyde levels during incubation periods. Therefore, catechin may be used as an antioxidant to reduce sperm abnormalities and improve sperm quality in boar semen [46]. On the other hand, results obtained by Tvrda et al. showed that quercetin, another natural flavonol, is able to prevent the decline in spermatozoa viability, functional activity, and antioxidant capacity [47].

Some of these compounds have been reported in fruit extracts from the berries of other endemic Chilean plants (*Berberis microphylla*, *Luma chequen*, *Luma apiculata*, and *Amomyrtus meli*), with antioxidant activity shown by DPPH radical bleaching and FRAP test [48]. In a previous study, we demonstrated the high performance of MT-Ex as an antioxidant in endothelial cells and as a cardioprotective agent which generates an antioxidant effect at concentrations in the range of picograms to micrograms of phenols [49].

Our studies of antioxidant capacity showed that although the domesticated murtilla samples were of different genotypes, all five presented antioxidant potential at similar concentrations, demonstrated by their EC50 values; similarities were found in their gradients and kinetics, but not in the concentrations of phenols detected by HPLC. Measurement of oxidative stress by the Luminol technique was sensitive to differences between the genotypes evaluated; however, the dispersion of the data represented by their biological replicates did not represent a substantial difference between the samples. The use of this technique to evaluate the pharmacokinetics of time and concentration determined through a mathematical model allows the behaviour of antioxidant activity to be assessed and understood exclusively under conditions of refrigeration (17°C) in the MR-A semen extender.

The superoxide anion is known to be a highly reactive molecule, unstable over time. It is one of the main stimulators of oxidative damage [50] and in conjunction with H_2_O_2_ is one of the initiators of lipid peroxidation in sperm [51]. In our study, we evaluated the antioxidant effect of cells previously treated with H_2_O_2_ as an oxidizing agent, which were subsequently washed to study the intracellular antioxidant potential of MT-Ex. We observed that the addition of MT-Ex to cells under oxidative stimulation by H_2_O_2_ significantly reduced the production of O_2_^−^. However, we observed that the addition of MT-Ex at EC50 concentrations reduced the population with high ROS production by more than 50%, while MT-Ex supplementation in cells without oxidative stimulation did not result in a reduction in the basal oxidation count. Thus, ROS reduction was intrinsically limited to the cells after induced stress, while the oxidative levels of cells with normal endogenous ROS production were not affected, suggesting that MT-Ex reduces the impact of H_2_O_2,_ when the cells already initiated a process of substantial ROS increase, indicating then that the scavenging effect of MT-Ex should be limited only under conditions where the sample presents a high endogenous production of ROS. However, due to the large conformation of the MT-Ex components, it is not possible to suggest a specific target different to H_2_O_2_ and O_2_^−^ in ROS reduction.

We did not observe a substantial reduction in lipid peroxidation by MT-Ex in sperm samples stimulated by FeSO_4_ (Fenton's pathway) or in samples in the basal condition. Studies in rat liver microsomes have shown that the application of high concentrations of flavonoids (75-100 *μ*M) in media supplemented with Fe^3+^ and EDTA favour the production of OH and therefore the triggering of lipid peroxidation; the concentrations of flavonoids applied in our assays were too low to stimulate endogenous production of ROS and induce peroxidation, as postulated by Laughton et al.'s study [52].

On the other hand, evaluation of the effect of MT-Ex on metabolic parameters showed a clear influence of the extract on the normal metabolism of spermatozoa under refrigeration conditions. An effect on MMP was observed, with a substantial reduction in the sperm count with basal MMP in samples without oxidant stimulation, while in spermatozoa under oxidative conditions, treatment with MT-Ex to reduce the oxidizing effect did not prove effective in stabilizing MMP.

Furthermore, we did not observe any regulatory or modulating effects of MT-Ex on succinate dehydrogenase by the MTT assay in either oxidant or antioxidant conditions. This enzyme (complex II of electron transport chain) is involved in the transformation of succinate to fumarate to generate an influx of electrons during the oxidative phosphorylation process in aerobic respiration [53]. We did not observe that any substantial change in enzyme capacity was mediated by the extract, at least in reduction of tetrazolium salt in formazan crystal, suggesting an independence of action on succinate dehydrogenase.

In the functional context, a study in isolated mitochondria reported important effects of the flavonoids quercetin, taxifolin, catechin, and galangin, which act as inhibitors of the respiratory chain of mitochondria or cause uncoupling [54]. Other authors report an important effect of ATP synthesis in bovine sperm, demonstrating the influence of quercetin, kaempferol, catechin, and epicatechin gallate in inhibiting ATPse/synthase. Quercetin generates an improvement in motility without modifying the ATP content and cellular respiration; however, motility and ATP production decrease after less than 2 hours [55]. We likewise found a substantial reduction of ATP in oxidant and antioxidant conditions. On this basis, we evaluated ATP production as a direct measure of metabolism functionality, especially as it is one of the adenosines involved in sperm motility. Depression of this adenosine is classically described as the effect of oxidative stress; if ROS are not quickly reduced by antioxidant enzymes, they lead to a decrease in the ATP content due to inefficient phosphorylation [56–58]. We observed that the ATP content was substantially decreased in the presence of H_2_O_2_ at concentrations of 122.7 *μ*M; however, the application of MT-Ex to sperm samples without ROS stimulation by H_2_O_2_ generated a significant reduction of ATP (>1 nm of ATP).

Some polyphenols, such as epigallocatechin gallate, epicatechin, quercetin, and kaempferol, have been associated with inhibition of ATP synthase complex (T0), occluding proton movement in subunits T0 and T1 [59]. Other studies have demonstrated the influence of flavonoids on complex I and cytochrome C; modulation of epicatechin, quercetin, and kaempferol is observed in these complexes, retarding H_2_O_2_ production [60].

It is not possible to generate an efficient balance when an oxidative process is triggered, and this could be directly related to sperm motility. Some studies suggest that the addition of catechin (50 *μ*M) could improve sperm motility in bovine spermatozoa [46]. In our study, we verified the effect of MT-Ex in boar sperm motility, suggesting that MT-Ex may have induced a reduction of the motile population. Interestingly, one of the chemical compounds of our extract contained catechin. Based on this, we suggest that there may be a synergistic/antagonistic or additive effect of the different components of the extract that is not necessarily present when the compounds are applied in isolation. The effect of the extract on sperm motility may well stimulate different pathways, blocking or activating the molecular machinery involved in sperm motility. In fact, we observed an important modulating effect in calcium movement, with a reduction in the intensity of the FLUO-4 AM indicator; this suggests inhibition/blocking of capacitating activation and reduction of the cAMP count.

Furthermore, it has been reported that polyphenols increase cell viability, decrease intracellular Ca^2+^ levels and ROS formation, and improve mitochondrial membrane potential in cells [61]; however, the mechanisms involved in this process are not completely elucidated, especially in the control of intracellular free calcium according to the time and concentrations of antioxidant treatment [62]. On the other hand, new reports have shown that quercetin (quercetin-3-O-glucoside) is able to interfere with different targets of cAMP signalling [63]. Our results suggest an influence of MT-Ex on calcium movement which may block hypermotility, cAMP-mediated capacitation, and the acrosome reaction, allowing the useful life of the boar sperm sample to be extended. In the same way, these parameters are involved as an important physiological indicator of fertility capacity, as well as the stability of the DNA [64]; in this sense, our results indicate a partial reduction in DNA fragmentation induced by oxidative stimulation and subsequent reduction of this status with MT-Ex, but without reaching the basal levels considered normal for the group without oxidative stimulation, indicating a differential index of protection, probably by the low concentration of MT-Ex reaching the DNA to reduce the oxidization of DNA and subsequent fragmentation, in comparison with the intra/extracellular milieu, supporting the idea of partial pharmacologic efficacy delimitated by the subcellular localization.

On the other hand, our results under long-term refrigeration showed beneficial effects on sperm viability and motility, with improved results in both parameters after long exposure times (168 hrs). This effect has been observed in long-term refrigeration using cysteine as an antioxidant, where motility and cell viability are substantially improved, while adverse effects were observed with tocopherol, which reduces mitochondrial potential, motility, and viability [65, 66]. Our initial results showed a reducing effect of mitochondrial parameters, reflected in the long incubation analyses; however, in contrast to the control, application of the extract led to a smaller loss of potential over time and reduced the production of oxidizing agents, suggesting a positive effect from the formulation of a seminal dose until the end of the analysis. In the incubation period, however, the endogenous production of total O_2_^−^ was not improved by the addition of the antioxidant, suggesting other oxidant species involved in the oxidization of sperm cell in the long term. Recent studies have shown that the glycolytic pathway is able to support ATP production and maintain the sperm in conditions to generate motility after the decoupling of oxidative phosphorylation [35, 67], so this regulation would allow greater efficiency in the refrigeration of boar semen.

## 6. Conclusion

The five genotypes of murtilla tested contain different phenolic compounds in different concentrations but present the same pattern of antioxidant activity. The antioxidant activity of MT-Ex controls the harmful prooxidant effect of H_2_O_2_ and reduces the impact of the oxidizing agent on DNA fragmentation; however, it also generates an important decrease in sperm metabolism, especially ATP production. This could directly affect the fertilization potential due to the reduction in sperm motility and the calcium pathway to activate capacitation and subsequent acrosome reaction. Nevertheless, we consider that the metabolic decrease and subsequent modulation of sperm capacitation are an advantage in extending the useful life of boar sperm samples refrigerated at 17°C.

## Figures and Tables

**Table 1:** Total polyphenol content (TPC, *μ*gGAE mL^−1^) in murtilla (Ugni molinae Turcz.) fruit aqueous extract obtained from genotypes INIA 14-4, 19-1, 22-1, 23-1, and 27-1. Phenolic compound concentration (*μ*g/mL) in murtilla (Ugni molinae Turcz.) fruit aqueous extract obtained from each genotype determined by HPLC analysis. Values are mean ± standard deviation.

	Genotype				
	14-4	19-1	22-1	23-1	27-1
TPC (*μ*gGAE mL^−1^)	85.5 ± 0.7	406.5 ± 0.2	314.5 ± 0.4	285.2 ± 0.3	264 ± 0.3
Gallic acid (*μ*g mL^−1^)	0.059 ± 0.02	0.013 ± 0.01	n.d.	n.d.	n.d.
Catechin (*μ*g mL^−1^)	2.696 ± 0.7	0.521 ± 0.7	10.015 ± 0.9	2.287 ± 1.1	1.812 ± 0.9
Quercetin-3-*β*-D-glucoside (*μ*g mL^−1^)	0.141 ± 0.1	3.211 ± 0.3	0.074 ± 0.01	0.131 ± 0.05	0.050 ± 0.02
Myricetin (*μ*g mL^−1^)	0.115 ± 0.001	0.026 ± 0.009	0.083 ± 0.004	0.119 ± 0.01	0.026 ± 0.000
Quercetin (*μ*g mL^−1^)	0.009 ± 0.001	0.471 ± 0.2	0.007 ± 0.002	n.d.	0.010 ± 0.001
Kaempferol (*μ*g mL^−1^)	0.014 ± 0.008	0.022 ± 0.006	0.035 ± 0.008	0.010 ± 0.001	0.025 ± 0.003

n.d.: not determined.

**Table 2:** Predicted EC50: predicted EC50 for each time analysed according to sigmoidal dose-response analysis.

Time (hrs)	EC50 MT-Ex G.14-4 (*μ*gGAE mL^−1^ **)**	SD
0.5	0.315	0.094
1	0.055	0.019
2	0.030	0.014
4	0.003	0.009
6	0.006	0.009

The data shown represent the effective concentration to reduce the oxidative stress by 50% at each hour evaluated.

**Table 3:** Effect of sperm motility on sperm samples with and without oxidant stimulation. The groups were “control”—sperm samples incubated in the MR-A semen extender, “oxidant”—sperm samples treated with oxidant agent, “MT-Ex”—sperm samples treated with 0.0315 *μ*gGAE mL^−1^ as antioxidant treatment, and “MT-Ex + oxidant”—sperm samples coincubated with oxidant agent and 0.315 *μ*gGAE mL^−1^ antioxidant treatment. Significant differences are represented for each parameter evaluated independently in treatment groups. Different letters correspond to significant differences between treatments but independently of group motility parameters.

Group	Control	Oxidant	MT-Ex	MT-Ex + oxidant
Static (%)	11.2 ± 1.5^a^	42.4 ± 1.3^b^	32.9 ± 1.2^c^	51.3 ± 1.2^d^
Motile (%)	89.6 ± 3.1^a^	56.3 ± 2.6^b^	65.3 ± 1.5^c^	46.3 ± 1.9^d^
Progressive (%)	25.6 ± 2.7^a^	5.3 ± 1.6^b^	21.1 ± 0.9^a^	2.4 ± 2.1^b^

## Data Availability

All data used to support the findings of this study are available from the corresponding author upon request.

## Supplementary Materials

Supplementary Materials Supplementary 1: composition of sperm TALP-HEPES and modification of the composition for each treatment. Basal TALP-HEPES maintains the basal condition of sperm samples without generating any physiological modification to induce capacitation. CAP.BUFFER includes 2.0 mM of CaCl_2_ for activating capacitation by the calcium pathway. MT corresponds to TALP-HEPES and 10 *μ*gGAE mL^−1^ of MT-Ex. MT + CAP.BUFFER contains the same characteristic of capacitating buffer but includes 10 *μ*gGAE mL^−1^ of extract to evaluate the calcium response in sperm samples.
