# Supplementary material for: Antioxidant Effect of a Polyphenol-Rich Murtilla (Ugni molinae Turcz.) Extract and Its Effect on the Regulation of Metabolism in Refrigerated Boar Sperm
Source: Oxid Med Cell Longev. 2019 Jun 3;2019:2917513. doi: 10.1155/2019/2917513 (PMC6589320; doi:10.1155/2019/2917513)
Supplement: Supplementary Materials — Supplementary 1: composition of sperm TALP-HEPES and modification of the composition for each treatment. Basal TALP-HEPES maintains the basal condition of sperm samples without generating any physiological modification to induce capacitation. CAP.BUFFER includes 2.0 mM of CaCl2 for activating capacitation by the calcium pathway. MT corresponds to TALP-HEPES and 10 μgGAE mL−1 of MT-Ex. MT + CAP.BUFFER contains the same characteristic of capacitating buffer but includes 10 μgGAE mL−1 of extract to evaluate the calcium response in sperm samples. [file 2917513.f1.pdf]

Supplementary Materials

Supplementary 1: Composition of sperm TALP-HEPES and modification of the composition for each treatment. Basal TALP-HEPES maintains the basal condition of sperm samples without generating any physiological modification to induce capacitation. CAP.BUFFER includes 2.0mM of $CaCl_2$ for activating capacitation by the calcium pathway. MT corresponds to TALP-HEPES and 10 µgGAE mL^{-1} of MT-Ex. MT + CAP.BUFFER contains the same characteristic of capacitating buffer but includes 10µgGAE mL^{-1} of extract to evaluate the calcium response in sperm samples.

**Reagent**	**BASAL: TALP-HEPES medium**	**CAP. BUFFER: TALP-HEPES + Calcium and BSA**	**MT + CAP.BUFFER: TALP-HEPES + Calcium, BSA and MT-Ex**	**MT: TALP-HEPES medium + MT-Ex**
**Glucose**	6 mM	5mM	5mM	6 mM
**NaCl**	113 mM	100mM	100mM	113 mM
**KCl**	2.7 mM	3.1mM	3.1mM	2.7 mM
**$CaCl_2$**	-	2.0mM	2.0mM	-
**$MgCl_2$**	0.5 mM	0.4mM	0.4mM	0.5 mM
**NaH_2PO_4**	0.4 mM	0.3mM	0.3mM	0.4 mM
**$NaHCO_3$**	20 mM	15mM	15mM	20 mM
**Pyruvate**	3,1 mM	1.0mM	1.0mM	3,1 mM
**Lactate**	20 mM	21.7mM	21.7mM	20 mM
**HEPES**	20 mM	20mM	20mM	20 mM
**BSA**	-	0.3%(wt/vol)	0.3%(wt/vol)	-
**mosm/kg**	290-300	290-300	290-300	290-300
**pH**	7.4	7.4	7.4	7.4
**Murtilla Extract (MT-Ex)**			0.315µgGAE mL^{-1}	0.315µgGAE mL^{-1}
